# Who sparks online flames? A personality-based investigation using scenario-based measures of flaming behavior

**DOI:** 10.3389/fpsyg.2026.1748827

**Published:** 2026-07-09

**Authors:** Ryohei Umetani, Naoki Konishi, Takuto Homma, Jin Kato, Nobuhiro Mifune, Takemasa Yokoyama

**Affiliations:** 1 Department of Information Technology and Human Factors, National Institute of Advanced Industrial Science and Technology, Tsukuba, Ibaraki, Japan; 2 Organization for the Strategic Coordination of Research and Intellectual Property, Meiji University, Suginami, Tokyo, Japan; 3 Faculty of Human Sciences and Cultural Studies, Yamanashi Eiwa University, Kofu, Yamanashi, Japan; 4 School of Economics & Management, Kochi University of Technology, Kochi, Japan

**Keywords:** dark triad, internet flaming, justice sensitivity, online aggression, online disinhibition, personality traits, risk-taking, social media behavior

## Abstract

**Introduction:**

With internet flaming emerging as a prominent social issue, strategies for addressing it are increasingly needed. Despite various countermeasures, the issue remains under-addressed due to a predominant focus on *post-hoc* responses rather than proactive interventions. This study explores personality traits that may be associated with a higher likelihood of initiating internet flaming, with the goal of providing preliminary insights that may help inform future preventive approaches.

**Methods:**

Two large-scale questionnaire surveys, with 3,040 participants in total, were conducted.

**Results:**

The findings provide insights into psychological factors that contribute to internet flaming and indicate trait patterns that tend to appear among individuals who play central roles in such incidents.

**Discussion:**

By examining psychological characteristics associated with internet flaming, this study offers an initial step toward building safer online spaces. While the predictive power of the models was limited, the results highlight potential directions for future research on personality-informed approaches to online conflict, complementing existing reactive strategies.

## 1 Introduction

The advent of the internet has profoundly transformed our lives, embedding online spaces into social and economic interactions. In particular, social networks have emerged as pivotal platforms not only for personal interaction but also for the dissemination of information and exchange of opinions. However, alongside the convenience brought about by these digital spaces, problematic behavior and defamation have become increasingly common, often leading to the escalation of contentious online debates, also known as “internet flaming” (Oksanen et al., 2022; Ray et al., 2024). In Japan, similar scandals continue to occur. For example, a major restaurant chain faced severe backlash after a customer posted a video of themselves licking a shared condiment bottle inside one of its outlets. The incident sparked widespread consumer disgust and distrust in the company's hygiene management. Immediately after the issue surfaced, the company's stock price fell by approximately 5%, resulting in an estimated loss of about JPY 16 billion in market capitalization (Magramo and Ogura, 2023). Thus, the challenges posed by this phenomenon persist and require continuous attention. As the internet continues to transform lifestyles and the social implementation of metaverse spaces, addressing these issues is critical (Banerjee and Kaur, 2023).

Post-incident management of online backlashes may present distinctive challenges in Japan's unique cultural context. A well-known Japanese proverb states, “The nail that sticks out gets hammered down,” reflecting a social tendency to discourage individuals who stand out. Previous research has shown that Japanese society is characterized by strong conformity to group norms (Fukushima et al., 2009; Nishikawa, 2025). These findings suggest that although deviant behavior may be less likely to occur in Japan, once it does occur, it may trigger especially strong backlash. Such behavior may be interpreted not merely as an individual act but as a violation of shared social expectations. In such a context, online backlash may be amplified by a collective motivation to sanction norm-deviant behavior and restore perceived social order. Against this background, examining online backlash in Japan from a psychological perspective-rather than relying solely on post-incident regulatory responses-represents a potentially valuable approach.

This study aimed to elucidate the personalities of individuals likely to engage in internet flaming behavior in order to curb this growing issue. Internet flaming is commonly defined as a hostile or aggressive form of internet communication (Kayany, 1998; O'Sullivan and Flanagin, 2003). Online backlash is related to concepts such as “trolling” and “toxicity.” Trolling is typically defined as intentionally posting provocative or aggressive content to incite conflict or disruption (Hardaker, 2010; Merriam-Webster, n.d.; Sanfilippo et al., 2018). Toxicity refers to hateful or aggressive speech and behavior in online contexts (Madhyastha et al., 2023). Prior research has also suggested that factors such as gender and ideology may shape engagement in these behaviors (Fichman and Akter, 2025; Fichman and Rathi, 2023; Fichman and Sanfilippo, 2015). This study did not focus on intentional and hostile communication toward specific others; rather, it focused on behaviors that can ignite internet flaming. It should be noted that the present study does not address the full range of behaviors involved in internet flaming itself. Rather, it focuses on ordinary social media posts that may unintentionally trigger internet flaming when they are perceived by others as provocative or norm-deviant. The scenarios used to assess flaming tendencies were intentionally limited to non-political and non-criminal situations and did not include many of the diverse forms of online conflict observed in real-world settings, such as political controversy, targeted harassment, or incidents involving explicit illegal acts. Accordingly, the findings of this study pertain specifically to reactions toward relatively low-stakes, ambiguous norm-deviant posts rather than to all forms of internet flaming. This focus reflects the cultural context described above. In Japan, many online backlash incidents do not necessarily stem from deliberate trolling intended to provoke others. Such intentional provocation may be discouraged by cultural norms. Instead, online backlash is often triggered by posts that deviate from social expectations without explicit intent to provoke or incite conflict.

This study offers an initial integrative exploration of personality characteristics associated with internet flaming. Previous studies have examined several personality traits that influence flaming and trolling on the internet. However, they have only demonstrated the influence of each personality trait in isolation. Examples include the dark triad, online disinhibition, low agreeableness and conscientiousness, and high extraversion (Andreassen et al., 2013; Buckels et al., 2014; Graham and Gosling, 2013). Recent research has analyzed the relationship between the Dark Triad and online trolling, revealing a consistently positive correlation between them (Hidalgo-Fuentes et al., 2025). A survey of actual vandals, rather than the personalities of individuals who commit vandalism, revealed several motivations for internet vandalism such as boredom, attention-seeking, and revenge (Shachaf and Hara, 2010). Studies analyzing the content of actual internet vandalism posts have identified three main characteristics of vandalism, including aggression, humor and success. However, most previous studies have examined only the effects of a single factor, trait, or aspect of personality. Because this approach does not allow researchers to control for a broad range of potentially relevant factors simultaneously, it remains difficult to determine which personality traits are most strongly associated with internet flaming behavior and the relative strength of their influence. Currently, a broad understanding of internet flaming behavior and a multi-dimensional perspective are lacking. Hence, this study aimed to identify personality factors associated with internet flaming behavior by adopting a broad view of the possible factors and controlling for the potential effects of each. To our knowledge, this study represents an early attempt to integrate previous fragmented research and open up a new theoretical direction.

Hypothesis: The positive association of Dark Triad and aggression with provocative norm-deviant self-presentation on social media would remain observable after controlling for other personality-related and demographic factors.

We examined newly hypothesized personality related traits, in addition to those identified in previous studies, that may be associated with internet flaming. Specifically, these newly hypothesized traits refer to justice-related tendencies, such as beliefs just-world and sense of fairness. We posited that one of the triggers of internet flaming—annoying and aggressive behavior toward others—represents not only an intention to harm but also an attempt to restore fairness. Belief in a just world refers to a cognitive bias in which individuals believe that the world is fundamentally fair (Lerner, 1980). It reflects a psychological tendency to assume that prosocial behaviors, such as effort and helping others, lead to positive outcomes, such as rewards or success, even when these outcomes are not directly related to the behaviors themselves. Conversely, it also involves the assumption that antisocial behaviors, such as moral corruption, negligence, or criminal acts, lead to negative outcomes, such as failure or punishment, even when these outcomes are unrelated to the behaviors themselves. Indeed, people who strongly beliefs in a just world are more likely to admire existing institutions and political leaders and have negative attitudes toward disadvantaged groups (Rubin and Peplau, 1975). Such people are also known to maintain their beliefs by attacking and blaming victims of incidents and accidents (Correia and Vala, 2003; Correia et al., 2007). In addition, people with strong beliefs in a just world are more likely to engage in prosocial behavior when they are offered prosocial actions, such as cooperation or assistance, but are also more likely to engage in negative behavior when they are offered the opposite, such as being robbed, beaten, or negatively treated (Umetani et al., 2020, 2023). Thus, we hypothesized that individuals with a high sense of fairness may be more likely to engage in extreme behavior when they perceive an unfair event, motivated by the need to compensate for their beliefs.

Currently, individuals, including influencers, can gain high visibility on social networks. Internet flaming may, in some cases, be driven by extreme attention-seeking behavior and gain an audience (Khamis et al., 2017; Marwick, 2015). In this case, we would expect less fear of flaming and less concern about being vilified by others, which may be associated with low media literacy and social desirability. Media literacy in the present study refers not to actual media literacy measured by objective indicators, but to subjective media literacy, that is, individuals' self-perceived beliefs about their own ability to understand, evaluate, and appropriately use media information. Against this backdrop, we explored the question of which type of personalities more likely to engage in internet flaming behavior. This study also examined the effects of restoring impartiality and media literacy on internet flaming behavior, which have not been examined in the past. This new perspective, including scholarly contributions, shows that internet flaming is not a uniform phenomenon driven by similar intentions and causes, but by a variety of factors.

Research question: To what extent are psychological and media-related factors, such as beliefs in just-world and media literacy, associated with the likelihood of engaging in provocative norm-deviant online self-presentation?

The present study was not designed to test a single specific theoretical model in which particular personality traits or factors are assumed to explain internet flaming. Rather, it aimed to provide an exploratory and integrative examination of a broad set of factors that have been suggested by previous research or newly derived as potentially relevant to internet flaming. The personalities measured to achieve the objectives of this study were diverse, and a large number of questions were asked. Therefore, the survey consisted of two studies: Study 1, whose purpose was to examine the validity of the flaming scenarios used to identify people likely to initiate flaming (hereafter “flaming protagonists”) and examine the personalities that influence the estimation of the flaming protagonists; and Study 2, that used the useful flaming scenarios and factors identified in Study 1 to further examine which personality characteristics are most likely to engage in flaming behavior.

## 2 Study 1

### 2.1 Methods

We conducted a questionnaire survey among the registered participants of a web research company MyVoice Communications, Inc. A simple arithmetic formula was presented midway through the questionnaire as an attention check, with the section explicitly instructing participants to not answer the items and leave them blank. Participants who followed these instructions and left the items unanswered were considered to have provided valid responses. Data were collected from 1,000 participants (50.0% male; mean age = 45.3 years). The study consisted of five separate surveys administered using a between-subjects design. Participants were assigned to only one of the five survey groups, with 200 individuals in each group; thus, no participant completed more than one survey.

In accordance with local legislation (Act on the Protection of Personal Information), ethical review was not required as no personally identifiable information was collected. All procedures were conducted in compliance with relevant ethical guidelines and regulations. This study was subject to an internal ethical pre-screening by AIST's Human Engineering Experimental Committee, which formally determined that the study did not fall under medical or life sciences research involving human subjects as defined by Japanese national guidelines (Reference Number: H2024-1471). The survey adhered to the principles outlined in the Act on the Protection of Personal Information and complied with JIS Q 15001 (Personal Information Protection Management System). All participants were informed that their participation was voluntary and that they had the right to withdraw at any time, with the assurance that their responses would be excluded from the dataset if they chose to withdraw.

### 2.2 Survey instrument

The questionnaire was divided into scenario evaluation and personality measurement sections. In all the questionnaires, the personality measurement section was placed after the scenario evaluation section.

#### 2.2.1 Scenario evaluation

The scenario evaluation section presented a fictitious social network posting scenario. All scenarios were presented in text only and were designed to simulate a situation in which Person A had posted the scenario. Participants were presented with plain text descriptions rather than a user interface resembling a specific social networking service. Nine scenarios were used: four flaming and five neutral. These scenarios were based on actual and flaming incidents. The criteria for selecting the controversial scenarios were that they should not involve political contexts or obvious serious crimes. Although the scenarios used in this study did not capture all aspects of the wide range of online controversies, they were selected to minimize the influence of context-dependent factors, such as political conflict.

The scenarios were evaluated by asking the participants about their reactions to Person A's post. The survey questions measured four reactions: discomfort, moral judgment, estimation of others' reactions, and the factor capturing behavioral intention and empathy, with two questions for each reaction, resulting in a total of eight questions. This scenario evaluation framework was specifically developed for this study to assess the appropriateness of the selected crisis scenarios using a multi-perspective approach while minimizing respondent burden. The following flaming scenarios were used:

1. Person A posted a video in which he tested whether a fire alarm would go off using a cigarette lighter in a private restroom of a convenience store.
2. Person A posted a video of himself dancing noisily inside a supermarket store that was open for business.
3. Person A, a real estate agent, posted under his own name that he served a celebrity couple and introduced them to a property worth JPY 400,000 per month.
4. Person A, a dairy farmer, posted a video of himself kicking livestock and engaging in other similar acts.

The following neutral scenarios were used:

1. Person A posted a story about how he returned to his hotel and took a shower after finishing his work on a business trip.
2. Person A posted that, on her day off, she went to a cafe that she had been curious about and found it to be a good refresher.
3. Person A posted that she watched the latest episode of her favorite anime and was so moved by it that she shed tears and expressed her hopes for the next episode.
4. Person A watched the latest episode of her favorite TV drama and posted that the episode was full of thrills and excitement.
5. Person A saw a popular movie in the cinema and was amazed by the visuals, music, and depth of the story.

Regarding scenario evaluation items, those measuring negative reactions to each scenario were as follows:

- How angry are you with Person A?
- How offended are you by Person A?
- How socially inappropriate do you think Person A's behavior is?
- How much do you think Person A's actions should be criticized?
- If Person A actually posted this, to what extent would you think it was flaming?
- If Person A actually posted this, to what extent would you think the public would criticize it?

Measurement items for the behavioral intention and empathy with Person A were as follows:

- How well do you understand Person A's thoughts and feelings?
- If you were in Persons A's situation, would you have been inclined to make the same post?

#### 2.2.2 Personality measurement

Based on the preceding discussion, a broad assessment of personality traits hypothesized to increase susceptibility to online backlash was necessary. However, because the inclusion of all relevant scales in a single questionnaire would have imposed excessive respondent burden, Study 1 employed a between-subjects design in which participants were randomly assigned to one of five groups. Each group completed a different subset of personality scales derived from prior theoretical and empirical considerations. Common demographic variables—age, gender, and marital status—were measured across all groups. The first group answered questions related to media-related measures. Specifically, the survey measured the frequency of using each mass media and social media (12 items), knowledge of media-related terms (7 items), subjective media literacy (5 items), media suspicion (5 items), and online disinhibition using the Multi-Dimensional Measure of Online Disinhibition (12 items). Questions regarding subjective media literacy and media suspicion were re-used from a previous study (Suzuki et al., 2023). For questions regarding the frequency of media use, Suzuki et al. (2023) had focused only on Twitter (now X); we, however, also included Instagram and TikTok as these social media sites are widely used in Japan. Words associated with media were digital tattoo, byte terror, buzz, fake news, echo chamber, personalized search, filter bubble, and “don't know the term.” The participants responded on a 5-point scale, with answers ranging from “I don't know the terms” to “I can explain the meaning of the terms to others.” Knowledge of media-related terms was intended to objectively measure media literacy. Frequency of media use was intended to measure the frequency of internet use, which was positively correlated with cyberbullying behavior. The other groups consisted mainly of scales measuring personalities considered more likely to engage in internet flaming behavior.

The second group completed a survey based on scales on the Dark Triad (12 items; Jonason and Webster, 2010; Tamura et al., 2015), the Interpersonal Reactivity Index (28 items; Davis, 1980; Himichi et al., 2017), the Balanced Inventory of Desirable Responding (24 items; Paulhus, 1991; Tani, 2008), and the Moral Foundation Questionnaire (20 items; Graham et al., 2011; Murayama and Miura, 2019). Empathy, which has been shown to be associated with internet flaming and trolling, was measured using the Interpersonal Reactivity Index, whereas morality was measured using the Moral Foundation Questionnaire. The survey given to the third group consisted of items related to beliefs in a just world (BJW; 12 items; Maes and Schmitt, 1999; Murayama and Miura, 2015), justice sensitivity (8 items; Baumert et al., 2014; Tham et al., 2019), and aggression (24 items; Buss and Durkee, 1957; Buss and Perry, 1992; Ando et al., 1999). To examine the impact of a sense of fairness on internet flaming behavior, which is the hypothesis derived in this study, we measured it using BJW and Justice Sensitivity. BJW is a measure of perceived fairness in a just world, whereas Justice Sensitivity measures attitudes toward unfair situations.

The survey in the fourth group consisted of the Promotional Prevention Focus Scale (PPFS; 16 items; Lockwood et al., 2002; Ozaki and Karasawa, 2011), Traditional Interdependent Self-Construal Scale (10 items; Hashimoto and Yamagishi, 2016), and the Domain-Specific Risk-Taking Scale (30 items; Blais and Weber, 2006). The PPFS was used to measure success, which is considered one of the characteristics of internet vandalism, as well as goal orientation toward gain proximity and loss avoidance (Summerville and Roese, 2008). The Traditional Interdependent Self-Construal Scale was used to assess individual differences in agreeableness, particularly in the interpersonal dimensions. While agreeableness is generally associated with prosocial tendencies across cultures, in the Japanese cultural context, it is often characterized by an interdependent and relational orientation rooted in mutual concern for others. Recent research has proposed that interpersonal agreeableness can be divided into two dimensions: harmony seeking and rejection avoidance. In line with this view, this study employed the Traditional Interdependent Self-Construal Scale to capture these two culturally relevant facets of agreeableness.

The fifth group consisted of items measuring the need for approval (18 items; Kojima et al., 2003), social dissatisfaction (5 items), the UCLA Loneliness Scale (10 items; Arimoto and Tadaka, 2019), and selective exposure (4 items; Suzuki et al., 2023). The items in this group measure the desire for what is considered a motivation for internet trolling, with the desire for approval measuring the desire for attention. Social dissatisfaction was used to measure the desire for revenge, in line with prior research, by assessing dissatisfaction toward various aspects of one's life and society—such as politics, personal relationships, income, mass media, and perceived social recognition—whereas the UCLA Loneliness Scale measured the degree of boredom. Social dissatisfaction was measured using five items adapted from the NIRA Basic Survey on Politics, Economy, and Society (NIRA Institute for Comprehensive Research Development, 2025).

Except for age and gender, all scenario evaluation items and scale items were measured using a five-point Likert-type scale. In the binomial logistic regression analyses, the dependent variable was coded as a binary indicator based on whether each scenario was evaluated as representing online flaming. Gender was treated as a categorical covariate, with female participants used as the reference category. The same coding and measurement procedures were applied in Study 2.

### 2.3 Results and discussion

We first conducted a confirmatory factor analysis to examine whether the scenario evaluation items functioned as intended. The results indicated acceptable model fit for all factors—discomfort, moral judgment, estimation of others' reactions, and the factor capturing behavioral intention and empathy—supporting the proposed four-factor structure. Accordingly, this structure was retained for subsequent analyses.

**Figure 1:**
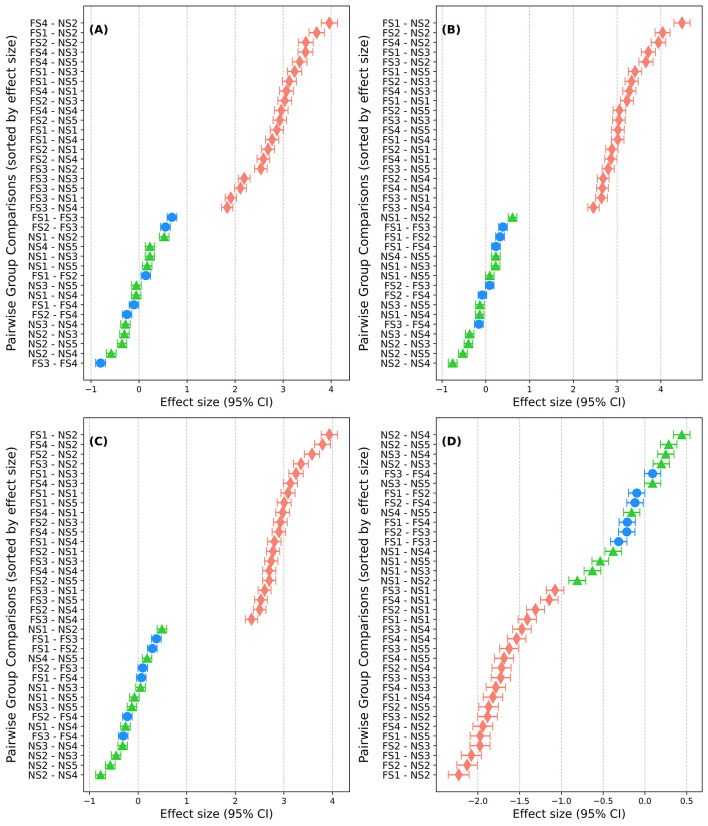
Effect size of scenario pair comparisons for each scenario by evaluation factor. This figure shows the effect size of each scenario-pair comparison in the analysis of variance, comparing the ratings of each scenario for each scenario rating factor. The vertical axis shows the pairs compared and ranked by effect size. The vertical axis labels indicate that FS represents the flaming scenario, and NS represents the neutral scenario. The horizontal axis represents effect size. Error bars indicate 95% confidence intervals. The colors of the plots indicate the differences between the pairs of scenarios: orange for flaming and neutral scenarios, blue for pairs of flaming scenarios, and green for pairs of neutral scenarios. Figure **(A)** shows the results of the discomfort measure, **(B)** the moral judgment measure, **(C)** the other person reaction estimation measure, and **(D)** the behavioral intention and empathy measure for Person A.

Next, we conducted a one-factor analysis of variance to determine whether the flaming scenarios elicited stronger negative emotional and moral evaluations than the neutral ones, as presented in the fictional social network posting condition. The dependent variable was the sum of each factor, and explanatory variable was each scenario. Significant differences were observed, likely owing to the large sample size of the analyzed data. Figure 1 shows the effect size of the paired comparisons for each scenario, confirming a significant difference between the flaming and neutral scenarios. The indicator used is Hedges' *d*-value.

The results indicate that, as expected, our flaming scenarios were judged to be morally unjustified, with more negative sentiments associated with them than with neutral scenarios. Therefore, we found all four flaming scenarios useful in this study and used them in Study 2.

Next, we conducted a binomial logistic regression analysis for each group to examine the personality traits more likely to cause internet flaming. The dependent variable was a factor measuring behavioral intention and empathy toward Person A of the scenario evaluation as the degree of susceptibility to generate internet flaming. The variable was constructed based on responses to eight questions: four questions corresponding to each of the four flaming scenarios, and two for each of the behavioral intention and empathy measurement items. Participants who rated any of the eight items 3 or higher on a 5-point scale were coded as 1, and all others were coded as 0. Regarding the proportion of the dependent variable, the number of 1s for the dummy variable out of 200 participants in each group was 83, 79, 75, 74, and 89, respectively, with the proportion for all groups being 40%. As expected, the distribution of behavioral intention and empathy scores for Person A in the flameout scenario was highly skewed, with most participants having low intention-empathy scores.

In the current phase of Study 1, we set the criterion of internet flaming loosely so that the weak association between a person likely to generate internet flaming and their personality would not be overlooked. For the independent variables, items based on previous studies were created according to the factor structure presented. All items created in this survey had a one-factor structure, except for media-related knowledge, which had a two-factor structure, that is, professional and general knowledge. Age, gender, and marital status were used as control factors. In this analysis, all independent variables related to personality were standardized. The results of the analysis showed significant differences for several factors in each group. The results are summarized in Table 1. The personality traits corresponding to the scales that showed significant differences were used in Study 2.

**Table 1:** Results of binomial logistic regression analysis for each group.

Group/predictors	β	SE	*p*	OR
*Group 1*				
Age	0.01076	0.0147	0.464	1.011
Gender (1 = Female)	−0.26591	0.339	0.433	0.767
Marital Status (1 = Married)	0.15615	0.3479	0.654	1.169
*Multi-dimensional measure of online disinhibition*				
Unique perspective on online environment	0.39956	0.1924	0.038	1.491
Change of alienation cognition	0.16504	0.1931	0.393	1.179
Change of relationship cognition	−0.0954	0.1833	0.603	0.909
*Frequency of media use*				
Mass media	−0.15999	0.2331	0.493	0.852
Social media	0.00217	0.2273	0.992	1.002
*Media-related knowledge*				
General knowledge	−0.16509	0.2265	0.466	0.848
Professional knowledge	0.35334	0.2121	0.096	1.424
Subjective media literacy	0.40583	0.2018	0.044	1.501
Media suspicion	−0.37437	0.2019	0.064	0.688
*Model fit*			*Nagelkerke R^2^ = 0.19*	
*Group 2*				
Age	−0.0115	0.0139	0.405	0.989
Gender (1 = Female)	−0.2752	0.3343	0.41	0.759
Marital status (1 = Married)	−0.1217	0.3589	0.735	0.885
*Dark triad*				
Machiavellianism	0.1047	0.2546	0.681	1.11
Psychopathy	0.1671	0.2644	0.527	1.182
Narcissism	0.7156	0.2155	<0.001	2.045
*Interpersonal reactivity index*				
Personal distress	−0.3585	0.2215	0.106	0.699
Empathic concern	−0.1844	0.2445	0.451	0.832
Perspective taking	0.2753	0.1888	0.145	1.317
Fantasy scale	0.1024	0.2022	0.612	1.108
*Balanced inventory of desirable responding*				
Self-deception	0.1296	0.2044	0.526	1.138
Impression management	−0.1331	0.1883	0.48	0.875
*Moral foundation questionnaire relevance subscales*				
Harm and fairness	0.306	0.2697	0.257	1.358
Ingroup, authority, and purity	−0.1792	0.2469	0.468	0.836
*Moral foundation questionnaire judgement subscales*				
Harm and fairness	−0.1307	0.2566	0.61	0.877
Ingroup, authority, and purity	−0.1584	0.2468	0.521	0.854
*Model fit*			*Nagelkerke R^2^ = 0.24*	
*Group 3*				
Age	0.00643	0.014	0.647	1.006
Gender (1 = Female)	−0.71068	0.3619	0.05	0.491
Marital status (1 = Married)	0.3026	0.3588	0.399	1.353
Victim	−0.1359	0.2444	0.578	0.873
Observer	0.01364	0.263	0.959	1.014
Beneficiary	0.48765	0.243	0.045	1.628
Perpetrator	−0.34212	0.2439	0.161	0.71
*Beliefs in a just world*				
Belief in ultimate justice	0.72516	0.248	0.003	2.065
Belief in immanent justice	−0.46756	0.2342	0.046	0.627
Belief in an unjust world	−0.27753	0.1989	0.163	0.758
*Aggression*				
Physical	0.45081	0.2178	0.038	1.57
Verbal	−0.12367	0.2441	0.612	0.884
Anger	0.27329	0.2306	0.236	1.314
Hostility	0.12839	0.1874	0.493	1.137
*Model fit*			*Nagelkerke R^2^* = 0.25	
*Group 4*				
Age	0.0203	0.0147	0.166	1.021
Gender (1 = Female)	0.9543	0.3705	0.01	2.597
Marital status (1 = Married)	−0.5909	0.3723	0.112	0.554
*Promotion/prevention focus scale*				
Promotion focus	0.3811	0.2227	0.087	1.464
Prevention focus	−0.5412	0.2358	0.022	0.582
*Traditional interdependent self-construal*				
Harmony seeking	−0.3086	0.2205	0.162	0.735
Rejection avoidance	0.2647	0.2352	0.26	1.303
*Risk-taking scale*				
Ethical	−0.1237	0.2623	0.637	0.884
Financial	0.0834	0.2344	0.722	1.087
Health/Safety	0.3094	0.2443	0.205	1.363
Recreational	0.6245	0.2555	0.015	1.867
Social	−0.3406	0.1797	0.058	0.711
*Model fit*			*Nagelkerke R^2^* = 0.22	
*Group 5*				
Age	−0.0161	0.013	0.216	0.984
Gender (1 = Female)	−0.8421	0.3357	0.012	0.431
Marital status (1 = Married)	0.3189	0.3589	0.374	1.376
*Need for approval*				
Praise seeking	0.1697	0.1691	0.316	1.185
Rejection avoidance	−0.0518	0.1698	0.76	0.949
Social dissatisfaction	−0.4562	0.1715	0.008	0.634
UCLA loneliness	0.4802	0.1833	0.009	1.616
Selective exposure	−0.2273	0.1635	0.165	0.797
*Model fit*			*Nagelkerke R^2^* = 0.18	

This table presents the results of binomial logistic regression analyses conducted separately for each of the five personality groups. Reported values include the estimated regression coefficients (β), standard errors (SE), *p*-values (*p*), and odds ratios (OR).

The objectives of Study 1 were to examine the validity of the flaming scenarios used to identify the flaming protagonists and explore the personality traits associated with internet flaming behaviors. As expected, negative reactions were observed because the flaming scenarios were based on incidents which actually stoked flames. Scores for intention and empathy toward the flaming protagonist, measured as the likelihood of engaging in flaming behavior, showed a very low mean. However, a small number of scores with a low mean but high variance was also observed. This suggests that internet flaming may occur when a small number of flaming posts generate strong antagonistic reactions toward them. The personality traits measured in this study were primarily those that were found to be associated with internet flaming in previous studies, although some of them showed no observable effects. While Study 1 divided the study sample into five groups owing to the large number of questions, these results may have proved difficult to detect in a study that examined only the effects of a single personality trait. Study 2 used the flaming scenarios presented in Study 1 and the scale on which significant differences were observed to show which personality traits have a greater effect on influencing the flaming protagonist.

Multicollinearity diagnostics indicated no serious multicollinearity problems in any of the five survey groups. Across all models, the maximum variance inflation factor (VIF) was 2.90, and the minimum tolerance value was 0.345.

## 3 Study 2

### 3.1 Method

We conducted a survey comprising 2,040 registered participants of the crowdsourcing service, Crowd Works Inc. Participants were provided with a written survey outline and consent form prior to the commencement of the survey, which they accepted. This constituted the provision of informed consent. The questionnaire included items measuring behavioral intention and empathy toward Person A in the four flaming scenarios created in Study 1, as well as items measuring personality traits for which significant differences were observed in Study 1. Sub-concepts were also included in the measurements. Personality measures included the Multi-Dimensional Measure of Online Disinhibition, subjective media literacy, dark triad, justice sensitivity, BJW, aggression, PPFS, Risk-Taking Scale, social dissatisfaction, and UCLA Loneliness Scale.

### 3.2 Results and discussion

The primary focus of this study was to explore which personality traits are associated with incendiary online behaviors, drawing on prior research that identified associations with such behaviors. Notably, some earlier studies have reported null effects. Accordingly, our hypotheses were partially supported. However, this study employed a multifaceted approach by dividing the participants into five distinct groups, a strategy deemed necessary because of the extensive number of questions. This methodological decision enabled the identification of significant results not evident in previous studies examining the relationship between a single personality trait with online aggression. Study 2 utilized the flaming scenarios from Study 1 and the scale on which significant differences were observed to demonstrate which personality traits had a greater effect on estimating the flaming protagonist.

Multicollinearity diagnostics were performed for the predictors included in the Study 2 regression model. The results indicated no serious multicollinearity problems, with the maximum variance inflation factor (VIF) being 2.81 and the minimum tolerance value being 0.356.

**Figure 2:**
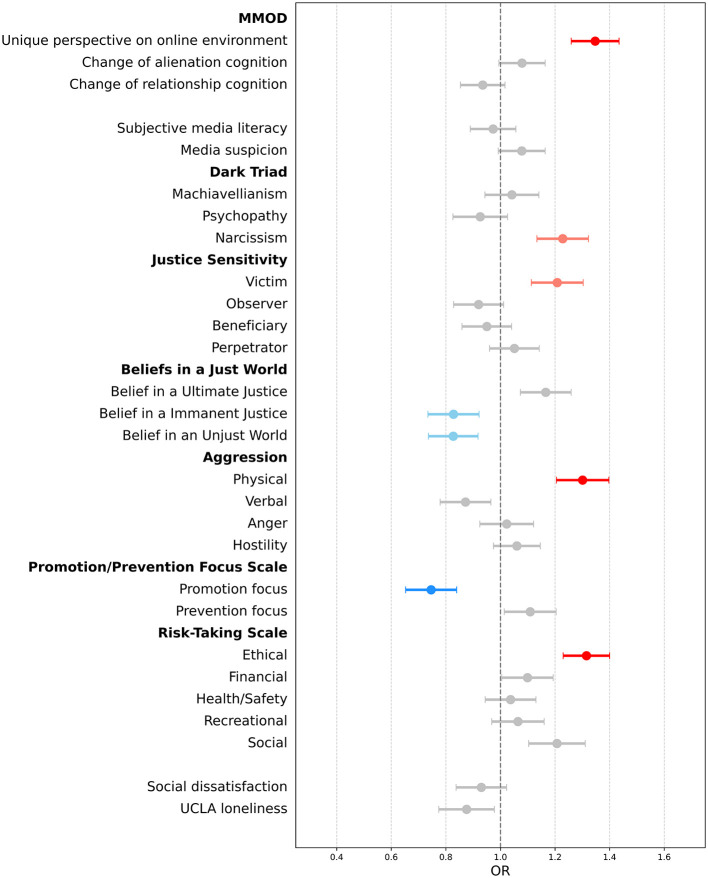
Influence of each personality trait on intention and empathy for flaming behavior. This figure illustrates the odds ratios derived from a logistic regression model, with error bars denoting standard errors. The factors on the vertical axis are arranged in descending order of odds ratios. The vertical axis represents the independent variable personality, whereas the colors of the plots denote the direction and magnitude of the effect. The factors for which no significant differences were observed are gray; values with p-values less than 5% and odds ratios greater than 1 are light red; values with odds ratios less than 1 are light blue; values with p-values less than 1% and odds ratios greater than 1 are dark red; and values with odds ratios less than 1 are dark blue. MMOD, Multi-Dimensional Measure of Online Disinhibition.

The results of the analysis indicated the presence of the following sub-concepts: online environment special regard (*p* < 0.01), a sub-concept of online disinhibition; narcissism (*p* < 0.05), a sub-concept of the dark triad; victim sensitivity (*p* < 0.05), a sub-concept of justice sensitivity; physical aggression (*p* < 0.01), a sub-concept of aggression; gain approach orientation (*p* < 0.01), a sub-concept of the PPFS; social risk taking (*p* < 0.01), a sub-concept of the Risk-Taking Scale; and ethical risk taking (*p* < 0.01), a sub-concept of the Risk-Taking Scale. Significant differences were observed for gain-approach orientation (*p* < 0.01); social risk-taking (*p* < 0.01); and ethical risk-taking (*p* < 0.05) (Figure 2 and Table 2). Furthermore, although several demographic variables showed significant effects within certain groups in Study 1, these patterns were not consistent across groups or between the two studies. Because of this lack of robustness, and because the demographic variables were treated as control factors rather than focal predictors, the present research does not further interpret these findings.

**Table 2:** Results of binary logistic regression analysis using predictors identified in study 1.

Predictors	Ave	SD	α	β	SE	p	OR
*Multi-dimensional measure of online disinhibition*							
Unique perspective on online environment	2.11	0.644	0.717	0.2982	0.0876	<0.001	1.347
Change in alienation cognition	2.99	0.764	0.724	0.0759	0.0849	0.372	1.079
Change in relationship cognition	3.58	0.825	0.754	−0.0674	0.0817	0.409	0.935
Subjective media literacy	2.77	0.708	0.847	−0.0274	0.0835	0.743	0.973
Media suspicion	3.36	0.563	0.532	0.0747	0.086	0.385	1.078
*Dark triad*							
Machiavellianism	2.22	0.777	0.807	0.041	0.0989	0.678	1.042
Psychopathy	2.51	0.647	0.566	−0.0772	0.1001	0.441	0.926
Narcissism	2.50	0.863	0.812	0.205	0.0944	0.03	1.228
*Justice sensitivity*							
Victim	2.90	0.950	0.601	0.1886	0.0951	0.047	1.208
Observer	2.80	0.871	0.774	−0.0829	0.0919	0.367	0.92
Beneficiary	2.38	0.869	0.744	−0.051	0.0909	0.575	0.95
Perpetrator	3.24	1.01	0.769	0.0495	0.0912	0.587	1.051
*Beliefs in a just world*							
Belief in ultimate justice	2.73	0.956	0.921	0.1533	0.0931	0.1	1.166
Belief in immanent justice	3.49	0.977	0.921	−0.1884	0.0938	0.045	0.828
Belief in an unjust world	3.70	0.846	0.864	−0.1902	0.0909	0.036	0.827
*Aggression*							
Physical	2.77	0.758	0.802	0.2628	0.0962	0.006	1.301
Verbal	2.97	0.693	0.771	0.0586	0.0859	0.495	1.06
Anger	2.76	0.873	0.844	−0.1368	0.0931	0.142	0.872
Hostility	3.02	0.710	0.817	0.0232	0.0986	0.814	1.023
*Promotion/prevention focus scale*							
Promotion focus	3.18	0.759	0.883	−0.293	0.0936	0.002	0.746
Prevention focus	3.28	0.739	0.862	0.1037	0.0952	0.276	1.109
*Risk-taking scale*							
Ethical	1.61	0.566	0.727	0.1884	0.1036	0.069	1.207
Financial	1.57	0.609	0.773	0.0361	0.093	0.698	1.037
Health/safety	1.73	0.545	0.593	0.0624	0.0961	0.516	1.064
Recreational	1.73	0.616	0.712	0.0947	0.0942	0.315	1.099
Social	3.19	0.575	0.562	0.2736	0.0851	0.001	1.315
Social dissatisfaction	3.53	0.562	0.518	−0.0728	0.0927	0.432	0.93
UCLA loneliness	2.95	0.812	0.897	−0.1323	0.1018	0.194	0.876
*Model fit*	*Nagelkerke R^2^ = 0.17*						

This table presents the results of the binary logistic regression analysis conducted in Study 2, using only the personality predictors that showed significant effects in Study 1. For each predictor, descriptive statistics—mean (Ave), standard deviation (SD), and Cronbach's alpha (α)—are reported, followed by the estimated regression coefficients (β), standard errors (SE), *p*-values (*p*), and odds ratios (OR).

## 4 General discussion

Before discussing the implications of these findings, it is important to clarify that the present study examined flaming tendencies using non-political, non-criminal, and non-targeted scenarios. These scenarios represent only a limited subset of real-world online flaming, and therefore the results should be interpreted as applying to this specific class of low-stakes, norm-deviant posts rather than to all forms of internet flaming.

This study systematically examined personality traits associated with tendencies related to internet flaming behavior. The results demonstrated patterns that differ in some respects from previous studies in terms of the personalities that were identified as likely flaming protagonists. The objective of Study 1 was to assess the validity of the flaming scenarios employed to identify potential flaming protagonists and narrow down the personality traits associated with flaming. Study 2 aimed to identify the personality traits that showed a more substantial association with internet behavior, leveraging the findings of Study 1. Consistent with previous studies, the present study found that the Dark Triad and online disinhibition were positively associated with internet flaming behavior, suggesting that these factors may contribute to individuals' engagement in such behavior (Buckels et al., 2014; Hidalgo-Fuentes et al., 2025; Lapidot-Lefler and Barak, 2012). It also revealed significant differences in personalities associated with attention-seeking, which is considered a motivation for internet flaming, and aggression and risk-taking, which are considered the main contents of flaming posts.

A significant discrepancy was identified in the BJW and justice sensitivity factors, which were measured to examine the hypothesis that annoyance and aggressive behavior toward others, originally derived as causes of internet flaming, act to restore fairness. The results of this study make a meaningful contribution to the literature by shedding light on how various personality traits are related to internet flaming behavior. Victim sensitivity, a subcategory of justice sensitivity, exhibited a positive effect on internet flaming behavior. Victim sensitivity is defined as sensitivity to losses due to unfair events. When individuals experience high sensitivity, they tend to avoid circumstances in which they could be subjected to treatment perceived as more unfair than that experienced by others. One possible interpretation is that this pattern may be partly attributable to the rise of influencers and other figures who have achieved economic and social success through high visibility on social media. The backlash scenarios used in this study did not involve direct attacks on specific individuals or groups but rather assumed diffuse or unspecified targets. Therefore, they did not represent corrective actions against personally experienced injustice. In this context, individuals high in victim sensitivity may frequently encounter highly visible figures online, which could motivate provocative engagement or attempts to elicit reactions from large audiences. However, although victim sensitivity showed a significant association with internet flaming behavior, the mechanism underlying this relationship remains unclear. The explanation offered above represents only one speculative possibility, as this study did not assess the motivational or cognitive processes that could account for this association. Therefore, the present results do not allow firm conclusions about why victim sensitivity is related to flaming behavior.

Contrary to findings related to justice sensitivity, this study's results indicate that BJW has a negative effect on internet flaming behavior. This outcome may be interpreted in light of the nature of these personality traits. belief in immanent justice (BIJ) and belief in an unjust world, which are subcategories of BJW, also exhibit negative effects on internet flaming. The factor structure of BJW is divided into time series in multilingual areas, but it is different in Japanese-speaking areas (Murayama and Miura, 2015). In the context of Japanese-speaking studies, belief in ultimate justice signifies the belief that a challenging present situation will ultimately be redressed by a favorable outcome, whereas BIJ denotes the conviction that wrongdoing will elicit a punitive response (Murayama and Miura, 2015). Consequently, individuals with a pronounced BIJ perceive incidents portrayed as flaming scenarios as malevolent acts that engender adversity for others and believe that they themselves will face retribution if they perpetrate such acts. Hence, the presence of BIJ may have a negative association with internet flaming behavior. BIJ may create psychological distance from aggressive or deviant behavior because it incorporates the belief that wrongdoing will ultimately be punished. However, because punishment expectancy was not directly measured in this study, this interpretation should be considered tentative. Meanwhile, belief in an unjust world is predicated on the notion that the world is replete with inequalities. This may be attributed to the ineffectiveness of the motivation to maintain fairness in driving behavior, as opposed to the impact of victim sensitivity within justice sensitivity.

An unexpected finding was that media-related knowledge, subjective media literacy, and media skepticism were not significantly associated with online backlash. This finding offers an important perspective. Online backlash is often assumed to stem from insufficient knowledge about the dynamics of online information dissemination. Previous research on cyberaggression has suggested that media literacy may reduce aggressive online behavior and related psychological processes. For example, media literacy components such as trust testing, privacy management, and cautious intimacy sharing with strangers have been shown to reduce both cyberaggression and moral disengagement, which in turn is associated with aggressive behavior online (Lee and Jun, 2024). However, these findings suggest that backlash behavior may not simply reflect careless actions due to ignorance; rather, in some cases, it may involve intentional engagement. These results imply that interventions focusing solely on improving media knowledge may be insufficient. Instead, greater emphasis on shared social norms, ethical standards, and awareness of potential social consequences may be necessary.

This study aimed to explore and identify the type of personalities that are more likely to generate internet flaming. While many studies have demonstrated the influence of individual traits on internet flaming, this study controlled for the influence of each trait. The study findings provide clues for mitigating internet flaming. Most respondents exhibited strong negative reactions toward Person A in the flaming scenarios, suggesting a pervasive latent dislike for those deviating from social norms. This finding underscores the notion that individuals' reactions to flaming are not contingent on the specific individual involved, but rather, are influenced by the act itself. Furthermore, the study suggests some consistency in the observed associations between certain personality traits and internet flaming, indicating that these traits hold a consistent association with flaming behaviors despite their sporadic manifestation. These findings suggest that identifying social networking service users' personality traits and types may offer a potential avenue for mitigating the occurrence of radical posts that give rise to internet flaming by implementing interventions tailored to individual characteristics.

A limitation of this study is that the flaming scenarios developed did not encompass all aspects of the diverse range of internet flaming behaviors observed in the real world. This restriction precluded a broad examination of internet flaming behavior itself, because the scenarios captured only a specific subset of ordinary social media posts that may trigger online backlash. Thus, the scope of this study is limited to potential antecedent behaviors of internet flaming, rather than the diverse forms of flaming behavior observed in real-world online conflicts. Additionally, it inferred the likelihood of individuals engaging in internet flaming based on their reactions to flaming scenarios, treating these reactions as a proxy measure for flaming behavior. Furthermore, this study treated responses to flaming scenarios as a proxy indicator of backlash behavior, inferring the likelihood of engagement in such actions. This approach was adopted to maintain a conservative design, given prior research suggesting that the proportion of individuals who actively contribute to backlash is likely small. The distribution of scenario evaluations in this study was indeed highly skewed. To avoid overlooking meaningful associations, a composite variable combining behavioral inclination and empathy toward the scenario was used rather than actual behavior. Similarly, the threshold for classifying proneness for backlash behavior was set relatively low in Study 1. Nevertheless, this remains an indirect estimation, and future research should examine flaming behavior at the behavioral level by assessing whether individuals actually engage in posts that may lead to flaming. Nonetheless, to our knowledge, this study represents an initial integrative exploration of personality characteristics believed to influence internet flaming behavior. Its findings contribute to a more nuanced understanding of internet flaming behavior by offering insights that can inform ongoing research efforts to prevent internet flaming. Additionally, there are design limitations regarding Study 1. Study 1 adopted an exploratory screening design in which participants were randomly assigned to one of five groups, each completing a different subset of personality scales. This design was chosen to reduce participant burden and to allow a broad initial search across a large set of candidate predictors. However, this approach also introduces certain limitations. Because each participant responded to only one subset of scales, estimating a single model including all candidate predictors within Study 1 was not possible. Consequently, the significance of any given predictor identified in Study 1 cannot be evaluated while simultaneously adjusting for the full set of traits measured in the other groups. This design feature may allow some predictors to appear significant due to variance shared with constructs measured in other groups, which cannot be statistically assessed in Study 1. Accordingly, the findings from Study 1 should be interpreted strictly as exploratory and hypothesis-generating rather than definitive. Study 2 addressed this limitation by measuring all candidate predictors within a single sample and estimating a unified model that allowed all predictors to directly compete with one another.

## Data Availability

The raw data supporting the conclusions of this article will be made available by the authors, without undue reservation.
